# The association of auditory integration training in children with autism spectrum disorders among Chinese: a meta-analysis

**DOI:** 10.1042/BSR20181412

**Published:** 2018-12-11

**Authors:** Ning Li, Ling Li, Guimei Li, Zhongtao Gai

**Affiliations:** 1 Department of Pediatric Health Care, Jinan Children’s Hospital of Shandong University, Jinan, Shandong 250022, China; 2 Department of Pediatrics, Shandong Provincial Hospital Affiliated to Shandong University, Jinan, Shandong 250022, China

**Keywords:** Auditory integration training, Autism spectrum disorders, Chinese children, Meta-analysis, Randomized controlled trials

## Abstract

Randomized controlled trials (RCTs) have reported an inconsistent relationship about the auditory integration training (AIT) in children with autism spectrum disorders (ASD) among Chinese. The current study was to investigate the efficacy of AIT for children with ASD compared with those in control group by using meta-analysis. Relevant trials published were identified by an electronic search of PubMed, CENTRAL, EMBASE, WanFang, CNKI, and SinoMed databases up to December 31, 2017. Outcome of interest included childhood autism rating scale (CARS), autism behavior checklist (ABC), intelligence quotient (IQ), and autism treatment evaluation checklist (ATEC). Standardized mean difference (SMD) with 95% confidence intervals (CIs) was calculated using a random-effect model. Thirteen RCTs with 976 children with ASD were included for analysis. The pooled SMD showed that children with ASD had significantly lower ABC scores [summary SMD = −0.58, 95%CI = −0.79 to −0.38] and ATEC scores [summary SMD = −0.75, 95%CI = −1.05 to −0.45] in AIT group compared with that in control group. The analysis of pooled statistics put forward AIT could increase the IQ score when compared with that in control group [summary SMD = 0.59, 95%CI = 0.41–0.77]. A negative association was found about CARS scores between AIT group and control group. No publication bias was found and no single study had essential effect on the pooled results. In conclusions, AIT can reduce the score of ABC and ATEC and can increase the IQ score among children with ASD in Chinese. Therefore, it is recommended for Chinese children with ASD to receive AIT.

## Introduction

Autism spectrum disorders (ASD) or autism refers to a wide range of related cognitive and behavioral disorders [1]. ASD are characterized by social and communication difficulties, alongside repetitive behaviors and special interests [2]. Data from previous publication [3] indicated that every 88 children may have one with ASD, it developed slowly and the cause of ASD cannot be completely determined. It also showed that approximately 50% of children with ASD had sensitive hearing phenomena; moreover, paranoid behavior and poor verbal were closely linked to auditory abnormalities [4]. However, therapies were developed to overcome the common auditory sensitivity changes in autistic patients and collectively referred to as auditory integration therapies. Auditory integration training (AIT), which was first developed in France in 1982 by Berard [5], was one of the therapies. AIT involves 10 h of listening to electronically modified music delivered by headphones during two half-hour sessions each day for 10 days. The AIT device uses filtering to dampen the peak frequencies to which the individual is ‘hypersensitive’ and delivers sounds modulated by random dampening of high and low frequencies and intensities [6].

Sinha et al. conducted three reviews and meta-analysis to assess the association of AIT and other sound therapies for adults or children with ASD [7–9]. However, in those results, they concluded that there is not sufficient evidence to support AIT use. At present, there are more and more related RCTs about AIT treatment for children with ASD in China. Nonetheless, for the treatment of AIT, there existed marked disparities among studies owing to the variable research designs and limited sample sizes. Accordingly, we performed a meta-analysis to estimate the effect of AIT treatment for children with ASD in Chinese.

## Method and materials

### Study design

We performed the present meta-analysis adhering to the Preferred Reporting Items for Systematic Reviews and Meta-Analyses (PRISMA) statements [10].

### Identification and selection of studies

We conducted a broad search of four databases, including PubMed, CENTRAL, EMBASE, WanFang, CNKI, and SinoMed databases, to identify relevant studies up to December 31, 2017. The following Mesh terms were used: “auditory integration training” OR “auditory therapy” AND “autism” OR “autistic children” OR “autism spectrum disorder” AND “Chinese”. Additional references were searched through manual searches of the reference lists and specialist journals. No language restrictions were applied.

To be eligible for inclusion in our study, publications had to meet all the following criteria: (1) study conducted with randomized controlled trials (RCTs); (2) reported the studies on Chinese children; (3) children with ASD are diagnosed using diagnostic manual of mental disorders (4th edn) (DSM-IV) [11] and international classification of diseases-10th (ICD-10) [12] or diagnosed using a standard diagnostic instrument; (4) AIT group was accepted additionally with AIT based on the treatment of the control group; (5) patients with ASD were Chinese children only; (6) reported outcomes of interest (i.e. childhood autism rating scale (CARS), autism behavior checklist (ABC), intelligence quotient (IQ), and autism treatment evaluation checklist (ATEC)); (7) availability of mean and standard deviation (SD) of scores about CARS, ABC, IQ, or ATEC.

Furthermore, children with impaired brain development, children with epilepsy and mental disorders, hyperemia and inflammation of the middle ear, deafness, and other abnormal hearing were excluded.

### Data extraction

Two investigators screened the titles and abstracts of potentially relevant studies. The same two reviewers retrieved the full text of relevant studies for further review. A third senior investigator resolved any discrepancies between reviewers. If reviewers suspected an overlap of populations in a report, they contacted the corresponding author for clarification; we excluded studies with a clear overlap.

The same pair of reviewers extracted study details independently. A third investigator reviewed all data entries. We extracted the following data: author, publications years, mean age or age range, diagnostic criteria, treatment method both for AIT group and control group, sample size, outcomes of interest and scores (mean ± SD) for each outcome. Meanwhile, trial validity assessment was done independently, and a trial quality assessment as assigned (A to C) according to the *Cochrane Reviewers’ Handbook* 4.2.2 [13].

### Statistical analysis

The relationship about AIT in children with ASD was pooled using standardized mean difference (SMD), which could control heterogeneity between different studies and some other influencing factors, with 95% confidence intervals (CIs) for CARS, ABC, IQ, or ATEC [14]. A random-effects model for the current meta-analysis was used. Subgroup analysis by diagnostic criteria was performed. Heterogeneity of pooled results was assessed using Cochran’s Q-test and the Higgins *I*^2^ statistic [15]. *P*_heterogeneity_ < 0.1 or *I*^2^ > 50% suggested significant heterogeneity among the included studies [16]. Meta-regression was used to assess the potential of important covariates to exert substantial impact on between-study heterogeneity [17]. Begg’s funnel plot [18] and Egger’s linear regression test [19] were conducted to verify publication bias, and a value of *P*<0.05 was considered statistically significant. A sensitivity analysis [20] by exclusion of one study at the time was performed to assess the stability of results and potential sources of heterogeneity. All statistical analyses were conducted using the STATA software, version 12.0 (STATA Corporation, College Station, TX, U.S.A.). Unless otherwise specified, all *P* values were two-sided.

## Results

### Study characteristics

**Figure 1:**
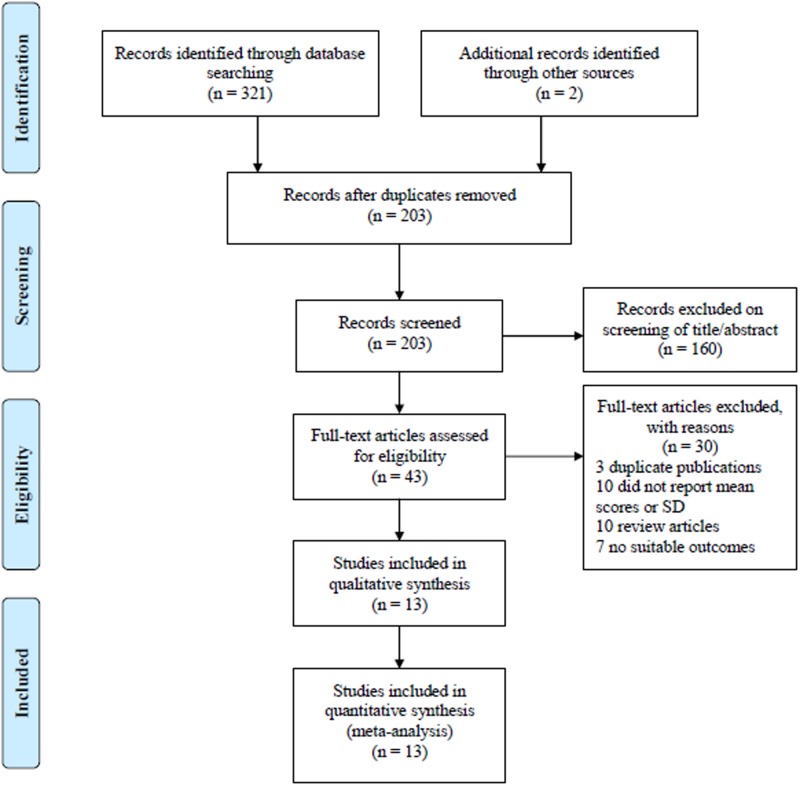
Selection process for clinical trials included in the meta-analysis

The flowchart of Figure 1 revealed detailed screening process of our analysis. In terms of the initial searching strategy, a total of 321 articles were obtained preliminarily. Additional 2 articles were identified through other sources. A total of 120 articles were removed after duplicated in different databases. After screening based on titles and abstracts, 160 articles were excluded. Subsequently, the full texts of remaining articles were carefully reviewed, and 30 articles of them were ruled out for the following reasons: 3 articles were duplicate publications; 10 articles failed to provide mean or SD of scores for outcomes; 10 articles were reviews; the other 7 articles had no suitable outcomes. Eventually, 13 RCTs [21–33] involving 489 children with ASD in AIT group and 487 children with ASD in control group were included in our meta-analysis. The main characteristics of the 13 RCTs are listed in Table 1.

**Table 1:** Characteristics of the included studies about auditory integration training in children with autism spectrum disorders among Chinese

Study, year [reference number]	Diagnostic criteria	Age (range or Mean ± SD)	Outcome	Quality	AIT group			Control group		
					*n*	Treatment method	Mean ± SD (scores)	*n*	Treatment method	Mean ± SD (scores)
Liu, Y.M. et al., 2015 [19]	DSM-IV	5.15 ± 0.94	CARS	A	20	Auditory integration training and individualized training	34.03 ± 4.64	25	No training	38.96 ± 3.95
Liu, Y.M. et al., 2015 [19]	DSM-IV	5.15 ± 0.94	ABC	A	20	Auditory integration training and individualized training	85.45 ± 8.01	25	No training	102.84 ± 20.97
Liu,Y.M. et al., 2015 [19]	DSM-IV	5.15 ± 0.94	IQ	A	20	Auditory integration training and individualized training	61.15 ± 16.89	25	No training	47.86 ± 13.52
Sun, Y.Y. et al., 2014 [20]	DSM-IV	3–9	ABC	B	22	Auditory integration training	73.77 ± 17.91	21	Music therapy	88.2 ± 18.37
Yu, D.M. et al., 2016 [21]	DSM-IV	3–7	ABC	A	40	Routine rehabilitation training and auditory integration training	84.1 ± 11.6	40	Routine rehabilitation training	86.5 ± 11.6
Wei, B.H. et al., 2012 [22]	CCMD-3	4–6	ATEC	B	43	Guided education training and auditory integration training	71.73 ± 8.49	43	Guided education training	78.69 ± 10.02
Xie, J.N. et al., 2014 [23]	ICD-10	2.5–5.5	ABC	B	47	Auditory integration training	50.89 ± 21.09	39	No training	67.03 ± 29.25
Wang, Y.J. et al., 2016 [24]	DSM-IV	4.07 ± 1.54	ABC	B	35	Routine rehabilitation training and auditory integration training	94.6 ± 13.17	35	Routine rehabilitation training	94.26 ± 10.91
Wang, Y.J. et al., 2016 [24]	DSM-IV	4.07 ± 1.54	CARS	B	35	Routine rehabilitation training and auditory integration training	38.66 ± 6.33	35	Routine rehabilitation training	39.57 ± 9.19
Wang, Y.J. et al., 2016 [24]	DSM-IV	4.07 ± 1.54	IQ	B	35	Routine rehabilitation training and auditory integration training	60.89 ± 16.52	35	Routine rehabilitation training	56.52 ± 17.23
Li, W.J. et al., 2013 [25]	DSM-IV	3–6	ABC	B	24	Routine rehabilitation training and auditory integration training	57.09 ± 11.72	26	Routine rehabilitation training	63.52 ± 10.1
Li, W.J. et al., 2013 [25]	DSM-IV	3–6	IQ	B	24	Routine rehabilitation training and auditory integration training	69.58 ± 12.39	26	Routine rehabilitation training	64.63 ± 12.01
Zhang, Y.H. et al., 2013 [19]	DSM-IV	5.6 ± 2.1	ABC	B	43	Comprehensive treatment and auditory integration training	68.3 ± 9.1	43	Comprehensive treatment	75.1 ± 11.6
Zhang, Y.H. et al., 2013 [26]	DSM-IV	5.6 ± 2.1	IQ	B	43	Comprehensive treatment and auditory integration training	67.1 ± 12.7	43	Comprehensive treatment	58.1 ± 14.2
Wu, Y.Z. et al., 2016 [27]	DSM-IV	3–9	ABC	B	45	Comprehensive treatment and auditory integration training	64.37 ± 9.25	45	Comprehensive treatment	73.68 ± 11.26
Wu, Y.Z. et al., 2016 [27]	DSM-IV	3–9	IQ	B	45	Comprehensive treatment and auditory integration training	69.43 ± 11.65	45	Comprehensive treatment	61.53 ± 12.17
Zhang, J. et al., 2014 [28]	DSM-IV	3–6	ABC	A	40	Routine rehabilitation training and auditory integration training	80.3 ± 10.26	40	Routine rehabilitation training	84.57 ± 11.36
Wang, J.H. et al., 2017 [29]	DSM-IV	2–8	ABC	B	32	Routine rehabilitation training and auditory integration training	64.59 ± 9.48	32	Routine rehabilitation training	74.14 ± 12.84
Wang, J.H. et al., 2017 [29]	DSM-IV	2–8	IQ	B	32	Routine rehabilitation training and auditory integration training	68.22 ± 12.48	32	Routine rehabilitation training	59.12 ± 13.29
Chen, W.H. et al., 2017 [30]	CCMD-3	2–6	IQ	B	48	Routine rehabilitation training and auditory integration training	67.42 ± 12.98	48	Routine rehabilitation training	59.15 ± 14.12
Ye, B. et al., 2014 [31]	CCMD-3	5–7	ATEC	B	50	Guided education training and auditory integration training	71.74 ± 8.5	50	Guided education training	78.7 ± 9.99

Abbreviations: ABC, autism behavior checklist; ATEC, autism treatment evaluation checklist; AIT, auditory integration training; CARS, childhood autism rating scale; CCMD-3, classification of Chinese mental disorders and diagnostic criteria-3th; DSM-IV, diagnostic manual of mental disorders (4th edn); ICD-10, International classification of diseases-10th; IQ, intelligence quotient; SD, standard deviation.

### Main result of AIT for the effect of ABC

**Figure 2:**
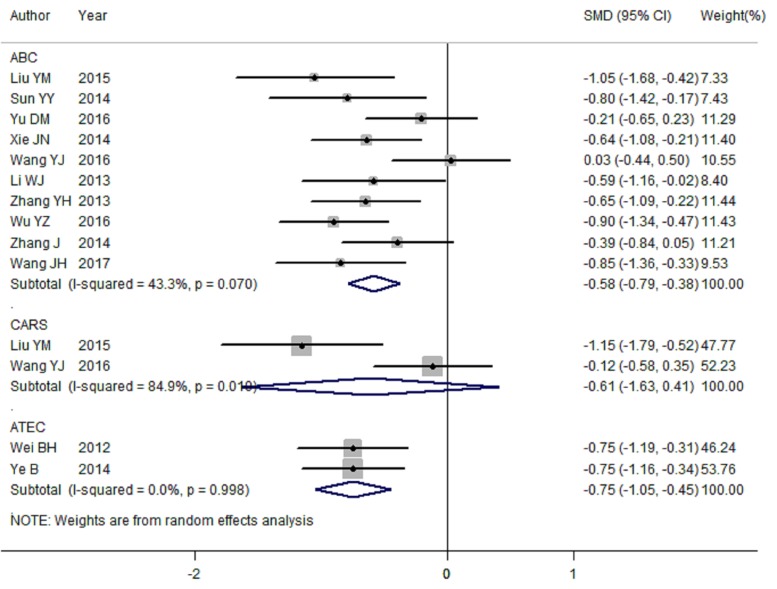
Meta-analysis of randomized controlled trials about ABC scores, CARS scores and ATEC scores for children with ASD among Chinese between AIT group and control group

There were 10 articles [21–23,25–31] included to assess the association about AIT for the effect of ABC among children with ASD. With significant heterogeneity (*I*^2^ = 43.3%, *P*_heterogeneity_ = 0.070), the analysis of primary pooled statistics put forward that children with ASD had significantly lower ABC scores in AIT group compared with that in control group [summary SMD = −0.58, 95%CI = −0.79 to −0.38] (Figure 2). Among the 10 articles, 9 of which were using DSM-IV diagnostic criteria, and only 1 article diagnosed ASD using ICD-10. Significant relationship was also found in the subgroup of using DSM-IV diagnostic criteria [summary SMD = −0.58, 95%CI = −0.81 to −0.35, *I*^2^ = 49.2%, *P*_heterogeneity_ = 0.046].

As availability of significant heterogeneity among pooled results, we then performed univariate meta-regression to explore whether the reason of heterogeneity was associated with covariates of publication year, case numbers and different diagnostic criteria. No significant contributions to between-study heterogeneity were found in this analysis (*P* = 0.341, 0.179, 0.521 for publication year, case numbers and different diagnostic criteria respectively).

To check the influence of each individual study involved in our meta-analysis on the pooled SMD for ABC scores, we removed studies in sequence. The results were not materially altered, suggesting that the pooled SMD were stable and robust.

Publication bias was assessed for ABC scores of AIT for children with ASD by Begg’s funnel plot and Egger’s linear regression test. In our meta-analysis, Begg’s test (*P*_r_ > |*z*| = 0.858) and Egger’s test (*P* > |*t*| = 0.289) show no obvious evidence of publication bias. As demonstrated in Figure 4, the symmetric shape of funnel plot supported the conclusion too.

### Main result of AIT for the effect of CARS

Two articles [21,26] were included to explore the association between AIT and the effect of CARS of children with ASD. The forest plot of analysis is illustrated in Figure 2. The scores of CARS were not significant in the AIT group when compared with the control group [summary SMD = −0.61, 95%CI = −1.63 to 0.41, *I*^2^ = 84.9%, *P*_heterogeneity_ = 0.010].

### Main result of AIT for the effect of ATEC

There are two studies [24,33] included to assess the scores of ATEC between AIT group and control group. As a result, the ATEC score was significantly lower in AIT group compared with those in control group [summary SMD = −0.75, 95%CI = −1.05 to −0.45, *I*^2^ = 0.0%, *P*_heterogeneity_ = 0.998] (Figure 2).

### Main result of AIT for the effect of IQ

**Figure 3:**
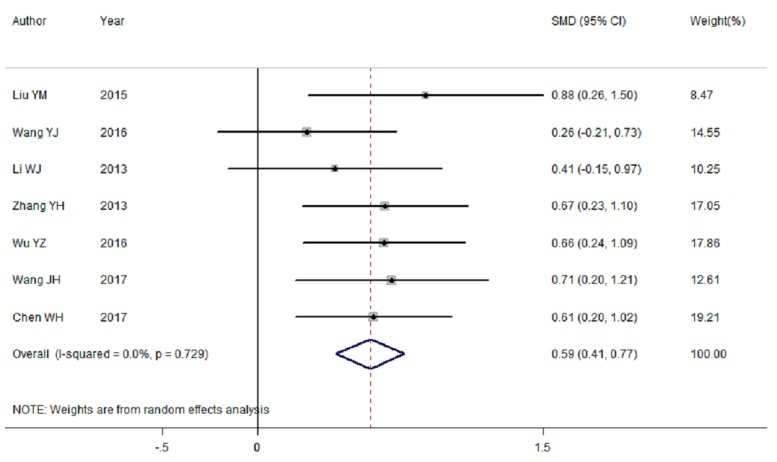
Forest plots of RCTs evaluating IQ scores for children with ASD among Chinese between AIT group and control group

Seven RCTs [21,26–29,31,32] were available to determine the effects of treatment on IQ in AIT group. The pooled result showed that the IQ score was significantly higher in AIT group than that in control group [summary SMD = 0.59, 95%CI = 0.41–0.77, *I*^2^ = 0.0%, *P*_heterogeneity_ = 0.729]. Figure 3 showed the forest plot of SMD with corresponding 95%CI about IQ scores between AIT group and control group. Six of the 7 RCTs were diagnosed by DSM-IV, and only 1 article using classification of Chinese mental disorders and diagnostic criteria-3th (CCMD-3) diagnostic criteria. A positive association [summary SMD = 0.59, 95%CI = 0.39–0.79, *I*^2^ = 0.0%, *P*_heterogeneity_ = 0.608] was found in the subgroup analysis of CCMD-3 diagnostic criteria.

**Figure 4:**
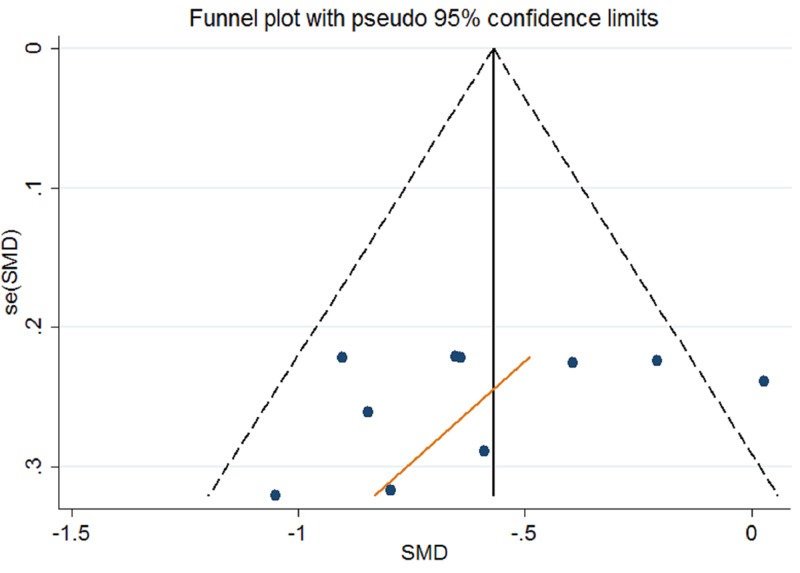
Begg’s funnel plots for assessment of publication bias about AIT for ABC scores

To investigate the influence of each individual study involved in the current analysis on the pooled SMD for IQ scores, we removed studies in sequence. The results were not materially altered, suggesting that the pooled SMD were stable and robust.

**Figure 5:**
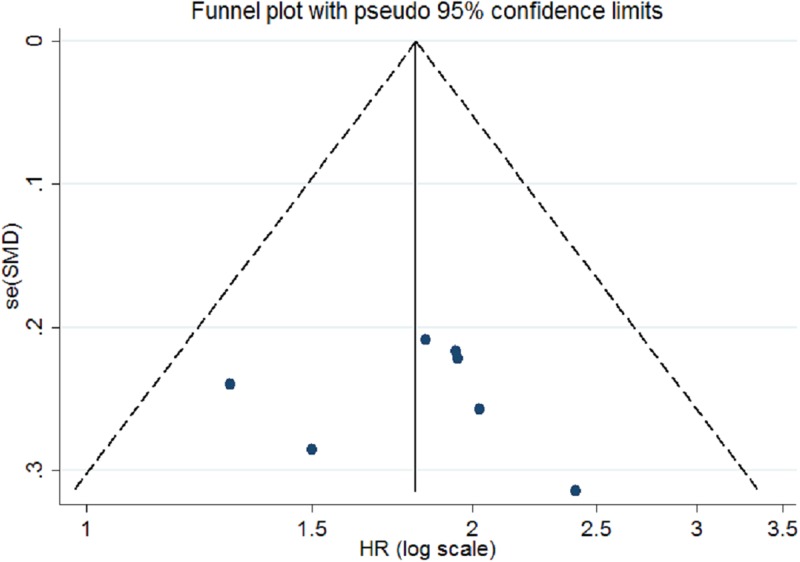
Begg’s funnel plots for assessment of publication bias about AIT for IQ scores

Publication bias was assessed by Begg’s funnel plot and Egger’s linear regression test. In our meta-analysis, Begg’s test (*P*_r_ > |*z*| = 0.368) and Egger’s test (*P* > |*t*| = 0.835) show no obvious evidence of publication bias. Figure 5 showed the funnel plot also supported the conclusion too.

## Discussion

In the present study, we explored the relationship between AIT and the effect of children with ASD using a meta-analysis. Findings from this meta-analysis indicated that children with ASD had significantly lower ABC scores and ATEC scores while received AIT. The result also found that IQ scores in AIT group were significantly higher than that in control group. Through our subgroup analyses, the results were consistent with the overall results.

In the previous study [8], negative association was found for adults or children with ASD while receiving AIT using six RCTs. Of their included studies, the results did not report the difference between AIT group and control group in a largest study [34]. A small trial inferred no long-term benefit for ASD when using the treatment of AIT [35]. However, one study obtained an increased in ABC scores in the AIT group at 3 months compared with that in control group [36]. In our meta-analysis, the ABC scores and ATEC scores were significantly lower and continued to decline in AIT group after a period of intervention, while the IQ scores were significantly higher in treatment conditions. Seven of our included studies [21,22,25,27–29,31] indicated that children with ASD had better status in AIT group in the fields of language, social interaction, physical movement, take care of themselves, mood, and sleeping using ABC rating scale. This illustrated that symptoms in children with ASD had improved obviously [37].

As seen in Figure 2, in our whole pooled results, significant between-study heterogeneity was appeared in ABC scores and CARS scores, which is common in meta-analysis [38]. We then performed meta-regression to assess this high heterogeneity with covariates of publication year, case numbers, and different diagnostic criteria. As a result, all the above-mentioned factors were not found to significantly contribute to heterogeneity. However, no individual study involved in our meta-analysis had essential effect on the pooled SMD for ABC scores and ATEC scores when we performed the sensitivity analysis. Furthermore, no publication bias was found in the study. There analyses results suggested that the pooled SMD were stable.

Our meta-analysis has the following advantages: first, we performed the first meta-analysis to expound the relation of AIT in children with ASD among Chinese and obtain a positive result. The results were not influenced by geographical area as we only included Chinese children with ASD. Second, according to our final pooled analysis for each individual study, larger participants of children with ASD were included. And this may strengthen the accurate comparisons between AIT group and control group. Third, no publication bias was found due to Egger’s test and funnel plot, which indicated that our results were stable across included studies.

There are several limitations need to be mentioned in the present study. First, although most of the diagnostic criteria of ASD were DSM-IV, different diagnostic criteria were existence in the included RCTs. This may affect the pooled results of SMD and 95%CI. However, sensitivity analysis did not support this opinion. Second, only two RCTs were conducted to assess the association for CARS scores and ATEC scores using the treatment of AIT, and we found a negative relationship in CARS scores. Hence, more related RCTs are wanted to confirm AIT in children with ASD in CARS and ATEC rating scale. Third, two RCTs with no training, 1 with music therapy, 6 with routine rehabilitation training, 2 with guided education training and 2 with comprehensive treatment were used in the control group. Different treatment of the control group could increase the heterogeneity between studies. In our meta-analysis, we found significant heterogeneity in the results of ALT for ABC scores. Therefore, different treatment of the control group may be an influencing factor on the significant heterogeneity.

In summary, AIT can reduce the score of ABC and ATEC and can increase the IQ score among children with ASD in Chinese. Therefore, it is recommended for Chinese children with ASD to receive AIT.

## Abbreviations

ABC: autism behavior checklist
AIT: auditory integration training
ASD: autism spectrum disorders
ATEC: autism treatment evaluation checklist
CARS: childhood autism rating scale
CI: confidence interval
IQ: intelligence quotient
SMD: standardized mean difference
